# Insecure attachment is associated with the α-EEG anomaly during sleep

**DOI:** 10.1186/1751-0759-1-20

**Published:** 2007-11-01

**Authors:** Eileen P Sloan, Robert G Maunder, Jonathan J Hunter, Harvey Moldofsky

**Affiliations:** 1 Integrated Medicine Project, Department of Psychiatry, Mount Sinai Hospital, Toronto, Canada; 2 Centre for Sleep and Chronobiology, Toronto, Canada; 3 University of Toronto, Toronto, Canada

## Abstract

**Background:**

The α-EEG anomaly during sleep, originally associated with chronic pain, is noted in several psychiatric and medical conditions and is also present in some normal subjects. The exact significance of the α-EEG anomaly is uncertain, but it has been suggested to be a nonspecific response to a variety of noxious stimuli. We propose that attachment insecurity, which is often associated with a state of hypervigilance during wakefulness, may be associated with the α-EEG anomaly during sleep.

**Methods:**

Thirty one consecutive patients referred to a Sleep Disorders Clinic for clinical assessment of sleep complaints underwent standard polysomnographic recording. The degree of alpha activity in polysomnographs was scored visually according to standard criteria. Attachment insecurity was measured with the Experience in Close Relationships – Revised questionnaire.

**Results:**

Attachment anxiety was significantly associated with the proportion of sleep in which α waves were present (df = 1, F = 5.01, p = 0.03). The relationship between the α-EEG anomaly and attachment anxiety was not explained by the distribution of sleep and mood diagnoses, medications, anxiety symptoms or depression symptoms.

**Conclusion:**

Interpersonal style in close relationships may be related to sleep physiology. Further research to determine the nature of the relationship between attachment, sleep and other factors that are related to each of these, such as a history of personal adversity, is warranted.

## Background

Alpha EEG activity, a sinusoidal rhythm with a frequency of 8–12 Hz, is the predominant EEG frequency recorded during passive wakefulness. It is recorded most prominently over the occipital and parietal regions of the scalp and is attenuated by eye opening and noise. During the onset to sleep, alpha EEG activity typically diminishes and is replaced by a low voltage activity of mixed frequency, mainly theta (4–7 Hz) and, as sleep deepens, delta (0.5–3 Hz) activity. The persistence of alpha activity during sleep (the α-EEG anomaly or alpha-delta anomaly) and its superimposition on delta waves, was initially described in patients with psychiatric illness [1] and subsequently in patients with fibromyalgia [2]. Many subsequent reports have found a similar association of the α-EEG anomaly with fibromyalgia [3-6] and with chronic "non-organic pain" [7]. The study of the α-EEG anomaly in the sleep of patients with chronic pain suggests that the anomaly represents an "intrusion" into normal sleep, i.e. that the anomaly acts as an indicator of a more vigilant state during sleep with resulting daytime symptoms of non-restorative sleep [8].

The α-EEG anomaly is not always associated with pain [9], and painful conditions are not always associated with the anomaly [10,11]. Rains and Penzien, examining the sleep records of over 1000 patients referred to a sleep disorders clinic, reported that the α-EEG anomaly occurred at a similar rate in patients with chronic pain, other medical/sleep disorders, and in psychiatric patients and that 60% of patients with the anomaly did not report pain [9]. The authors concluded that the α-EEG anomaly may reflect a non-specific response to a variety of noxious stimuli. Why some patients would show such a response to noxious stimuli when it is absent in others exposed to the same stimuli is unclear, but we postulate that psychological factors could play a role.

A body of recent research demonstrates that adult attachment style is associated with many aspects of health, health behaviour and disease. We have described a model of several causal pathways by which attachment insecurity may contribute to physical illness [12]. The intensity and duration of behavioural and physiological responses to stress, for example, may be moderated by attachment style. Attachment insecurity may also be associated with lower levels of vagal tone, which has implications for rapid recovery from periods of stress or arousal [13]. Furthermore, insecure attachment may increase perceived stress and make it difficult for the individual to use proximity to trusted others to buffer stress or recover from stressful events [14,15].

Attachment was first described by Bowlby as an interactional system that allows a child to maintain protective proximity to a care giving adult through the developmental period in which the child cannot care for him or herself [16]. Attachment plays an important role in stress regulation in the early years of life by facilitating perceived and actual security through a system of cues and responses that enable the infant to respond to perceived dangers and undesirable isolation with responses that increase proximity to its mother or primary caregiver. Individual attachment styles are recognizable clusters of trait-like interpersonal characteristics, designated in childhood by the categories secure, anxious, ambivalent and avoidant [17], including tendencies toward greater or lesser expression of distress, and greater or lesser preference for contact and proximity. Childhood attachment style is associated with affect regulation and, specifically, the capacity to be soothed and to feel subjectively secure [18]. Longitudinal studies demonstrate the stability of attachment style from childhood to early adulthood [18,19], and over decades in adulthood [20], but also the conditions under which attachment style may change with experience [19-21].

Self-report measures of adult attachment style measure dimensions of attachment anxiety and attachment avoidance [22]. According to Bartholomew's influential model of adult attachment [23], secure attachment is recognized as the combination of relatively low attachment anxiety and low attachment avoidance. Depending on the instruments used, about 50–60% of adults have a secure attachment style [24], which can be characterized as a flexible balance between preferences for autonomy and intimacy. Therefore, insecure attachment in its various types (indicated by high attachment anxiety, high attachment avoidance, or both) is common.

Individuals who are high in attachment anxiety are described as hypervigilant with regard to both the perception of possible threats and the availability and responsiveness of others [25]. Since insecure attachment is thought to be characterized by stress susceptibility and hyperarousal, it is plausible that the α-EEG anomaly during sleep is a marker of the unusual arousal associated with attachment insecurity expressed as a heightened responsiveness to a variety of noxious stimuli. In particular, if the α-EEG anomaly is associated with insecure attachment, then it would not be confined to patients with chronic pain or with any particular medical or psychiatric diagnosis. We hypothesized that the α-EEG anomaly would be found more often in people with higher levels of attachment insecurity and tested this hypothesis in a clinical sample of patients referred to a sleep clinic for sleep studies.

## Methods

This study was performed in a Sleep Disorders Clinic staffed by psychiatrists, respirologists and neurologists. Patients are referred to the clinic for a variety of clinical complaints, including snoring, insomnia, and excessive daytime sleepiness, typically by their primary care physician. Consecutive patients were approached to participate in the study. All subjects approached agreed to participate and provided informed consent. This study was approved by the Research Ethics Board at Mount Sinai Hospital, Toronto.

Subjects underwent a standard clinical polysomnographic (PSG) recording: surface electrodes were applied to the scalp at locations C3, C4 and Oz referred to A1 and A2, according to the International 10–20 system of electrode placement, using a low impedance paste. Electrodes were also applied to the submentalis muscle to record the electromyogram; bilaterally on the anterior tibialis muscle to record leg movements, bilaterally on the outer canthus to record the electro-oculogram; and in the second intercostal space at the midline bilaterally to record a two lead electrocardiogram. Respiratory effort was recorded by inductance plethysmography via belts places placed on the chest and abdomen. Arterial oxygen saturation was recorded with continuous recordings with a pulse oximeter, which uses a probe attached to the index finger. Airflow was measured via flow sensitive nasal prongs. A sleep diagnoses was made by a sleep specialist (board certified) or a sleep physician following assessment of the PSG and clinical assessment of the patient.

Recordings were carried out on the Sandman 6.1 PSG system or the CompuMedics Profusion PSG system 2.02. All subjects provided a minimum of 6 hours of data. The PSG was scored and staged visually by a trained technologist, blind to the patient's attachment style, according to standard criteria [26]. The technologist had been trained in the visual assessment and rating of α-EEG activity in the PSG. She had been part of a previous study on inter-rater reliability for the visual scoring of the α-EEG anomaly. The findings demonstrated a favourable inter-rater reliability [27]. Alpha activity was defined as lying within the range of 7–12 Hz, with a minimum peak to peak amplitude of 5 uV. It was rated according to the percentage of alpha events per epoch of stage 2 non-rapid eye movement sleep and slow-wave sleep. Arousal events and artifacts were eliminated from the analysis. After scoring each epoch, average percentage-of-alpha rating values were obtained for stage 2 non-REM sleep and SWS across the recording. There was no differentiation between tonic and phasic alpha activity. Rather than rating alpha activity as a continuous variable, we employed the method used in our clinical setting, i.e. the percentage of alpha activity noted in the overall recording in 5 groups: 0–20%, 20–40%, 40–60%, 60–80%, 80–100% [27]. Due to small numbers of subjects at the extremes of this range (0 – 20%, n = 2; 80–100%, n = 2), these groups were collapsed into three groups for this analysis: 0 – 40%, 40–60%, 60 – 100%.

Attachment was measured with the Experience of Close Relationships – Revised (ECR-R) questionnaire [22,28]. The ECR-R is a 36-item self-report questionnaire that surveys attitudes towards close relationships with intimate partners. Each statement is scored on a 7-point scale ranging from strongly disagree through neither agree nor disagree to strongly agree. The ECR-R has been derived through the application of item-response theory to choose the 36 best items from a pool of 323 attachment items drawn from the available and commonly used attachment instruments, all completed by 1,086 undergraduate students [22,28] and its reliability and validity are established [29]. Symptoms of anxiety and depression were measured with the Symptom Check List (SCL)-90-R [30].

In order to test the hypothesis that attachment insecurity is associated with the α-EEG anomaly, the relationship between ECR-R scores and α-EEG class was tested by univariate analysis of variance (ANOVA). The relationship between anxiety and depression subscales of the SCL-90-R and α-EEG class was also tested by ANOVA. Since neither anxiety nor depression symptoms differed by α-EEG class these variables were removed from the reported results to maximize degrees of freedom. Because attachment anxiety and attachment avoidance were significantly inter-correlated (R = 0.45, p = 0.01) the relationship of these variables to α-EEG class was tested in separate ANOVA.

## Results

Thirty-one sleep clinic patients participated in the study. Twenty five (80.6%) were female. Subjects ranged in age from 25 to 60 (mean 41.6, standard deviation 9.5). The distribution of subjects by percentage of the sleep study occupied by alpha activity was: 0 to 40% alpha – 9 subjects (29.0%), 41 to 60% alpha – 14 subjects (45.2%); 61 to 100% alpha – 8 subjects (25.8%). Clinical diagnoses, along with the medications patients were taking at the time of the study (Table 1) were similar in the three alpha groups. Subjects with clinical diagnoses of fibromyalgia (n = 6) or mood disorder (n = 13) did not differ from the remaining subjects with respect to α-EEG rating (Chi-square = 2.14, p = 0.44), mean attachment anxiety (p = 0.60) or attachment avoidance (p = 0.80). There were no significant differences between the α-EEG groups in depressive (df = 1, F = 0.04, p = 0.84) or anxiety symptoms (df = 1, F = 1.17, p = 0.29) as rated by the respective sub-scales of the SCL-90-R. Attachment anxiety was not significantly correlated with symptoms of anxiety (R = 0.19, p = .38) or depression (R = 0.04, p = 0.84). Neither was attachment avoidance significantly related to symptoms of anxiety (R = 0.01, p = 0.95) or depression (R = 0.27, p = 0.22).

**Table 1:** Comparison of groups defined by degree of alpha EEG intrusion into sleep^1^

	**Percentage of sleep study with α-EEG activity**		
	**0 – 40%**	**40 – 60%**	**60 – 100%**
	(n = 9)	(n = 14)	(n = 8)
Age (mean ± SD)	36.8 ± 11.9	43.8 ± 8.6	45.4 ± 9.0
Female gender	6 (66.6%)	12 (85.7%)	8 (100%)
**Clinical Diagnoses^2^**			
Psychophysiological insomnia	1 (11.1%)	0 (0.0%)	0 (0.0%)
Non-organic insomnia	2 (22.2%)	3 (21.4%)	2 (25.0%)
Idiopathic hypersomnia	2 (22.2%)	0 (0.0%)	0 (0.0%)
Obstructive sleep apnea	2 (22.2%)	0 (0.0%)	1 (12.5%)
Periodic limb movement			
Disorder/Restless legs syndrome	2 (22.2%)	5 (35.7%)	3 (37.5%)
Insomnia due to a mental disorder	2 (22.2%)	7 (50.0%)	4 (50.0%)
Fibromyalgia	1 (11.1%)	3 (21.4%)	2 (25.0%)
**Medication**			
No medication	6 (66.6%)	9 (64.3%)	4 (50.0%)
Antidepressant	1 (11.1%)	2 (14.3%)	2 (22.2%)
Benzodiazepine	0 (0.0%)	1 (7.1%)	0 (0.0%)
Opiate	1 (11.1%)	2 (14.3%)	0 (0.0%)

Other medication	2 (22.2%)	2 (28.6%)	2 (25.0%)

^1^All between-group differences are non-significant by ANOVA (age) or Kruskal Wallis test (categorical variables).

^2^Co-morbidities result in total percentages > 100%.

Attachment anxiety was significantly associated with α-EEG class (df = 1, F = 5.01, p = 0.03). If age is added to this analysis, it is not associated with α-EEG (df = 1, F = 1.33, p = 0.26), and the contribution of attachment anxiety to α-EEG remains significant (df = 1, F = 4.19, p = 0.05). If anxiety symptoms are included in this analysis, they are not associated with α-EEG class (df = 1, F = 0.88, p = 0.36), but the contribution of attachment anxiety to α-EEG class is somewhat reduced (df = 1, F = 3.64, p = 0.07). Attachment avoidance was not significantly associated with α-EEG class (df = 1, F = 2.73, p = 0.11).

**Figure 1:**
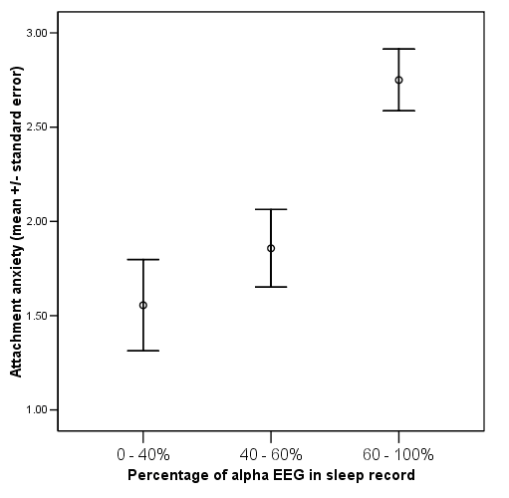
Magnitude of difference in attachment anxiety between groups low, medium and high in α-EEG anomaly during sleep.

In order to determine if the between-group differences in attachment anxiety were clinically meaningful, mean attachment anxiety score was calculated in each of the three α-EEG class groups. Compared to subjects with 0 to 60% alpha, in subjects with 61 to 100% alpha, attachment anxiety is greater by > 1 point on the 7-point scale of the ECR-R (Figure 1).

## Discussion

This examination of the relationship between attachment style and the degree of α-EEG sleep in a clinical population suggests that attachment insecurity, in particular insecurity expressed as anxiety about intimate relationships, is associated with a biological measure of sleep disturbance. The finding is not due to a higher degree of anxiety or depressive symptoms in anxiously attached individuals, which makes it unlikely that attachment anxiety is a proxy for generalized anxiety or depression. The link that is found between attachment insecurity and the α-EEG anomaly during sleep is consistent with the thesis that the anomaly is a marker of hyperarousal or hypervigilance which increases individual sensitivity to noxious stimuli during sleep.

The magnitude of the difference in attachment scores between α-EEG groups (Figure 1) is likely to be clinically meaningful. For example, we have previously measured ECR-R attachment anxiety scores (which range from 1 to 7) in hospital healthcare workers (a non-clinical sample): mean 2.48 ± S.D. 1.28 [31], healthy primary care patients: 2.42 ± 1.38 [13], outpatients with ulcerative colitis: 2.46 ± 1.34 [32], outpatients with heart failure: 2.49 ± 1.42, and emergency department patients: 3.29 ± 1.18 [33]. The level of attachment anxiety in sleep clinic patients, at 3.82 ± 1.13, appears to be higher than is found in non-acutely ill people and the difference between groups (> 1 ECR-R point) is a difference of about 0.75 standard deviations.

It is plausible that people who are high in attachment anxiety, likely as a result of earlier adverse experiences, and who are characterized during wakefulness as hypervigilant, anxious, and difficult to soothe, do not down-regulate this arousal state completely when they fall asleep. Conceptually, attachment anxiety is related to expectations of abandonment. It can be speculated that this expectation may be particularly salient, developmentally and in the context of the evolutionary purpose of the attachment system [34], when an individual must fall asleep and trust that their environment will remain secure and that their caregivers will remain present while they are unconscious. The usual mechanisms for ensuring proximity to the attachment figure, such as watching, crying, following, and clinging [35], are not available during sleep. The inevitable loss of control over care-seeking behaviors which occurs in sleep may lead individuals who are high in attachment anxiety to experience heightened arousal during sleep.

The lack of association between attachment avoidance and the α-EEG anomaly may be because attachment avoidance is not linked to hypervigilance during sleep or may be due to a lack of statistical power to detect a relationship in this small sample. The latter explanation is supported by the direction of the trend towards a relationship between these variables. To illustrate this trend, attachment avoidance was higher in subjects with 60 – 100% α-EEG (4.49 ± 1.17) than in subjects with < 60% α-EEG (3.19 ± 1.08, p = 0.02).

This study is limited by its small sample size and the use of a clinical convenience sample with mixed sleep diagnoses. The clinic in which the study took place is unique in that its multidisciplinary staff (psychiatry, respirology, neurology) attracts a more diverse range of clinical problems than are found in many sleep clinics (particularly, fewer patients with obstructive sleep apnea). This is not a major limitation, however, because the anomaly is not confined to any particular sleep diagnosis. We were unable to control what medications patients were taking at the time of their sleep study, including medications which affect sleep architecture. However, the range of medications was similar across the three groups and most patients (61%) were not taking any medication. In the clinical setting, only one polysomnograph was performed and patients did not typically undergo an adaptation night. As a result, we are not able to comment on the night to night stability of the α-EEG anomaly. Since many EEG phenomena are sensitive to the first night effect, further studies should incorporate at least two consecutive nights of polysomnograph recording. Finally, while we are confident in our technique of scoring the α-EEG anomaly, the use of quantitative EEG analysis would permit more precise analyses. Thus, we present findings that require replication in studies which address these limitations.

Further research on non-clinical subjects is required to determine if insecure attachment is associated with the α-EEG anomaly in the absence of sleep complaints. A larger study could test the prediction that α-EEG is most elevated in persons with the fearful attachment style (both high attachment anxiety and high attachment avoidance) as would be expected if α-EEG is determined in part by early adversity. It would be intriguing to explore the relationship between attachment insecurity and impaired sleep quality (chronic insomnia or non-restorative sleep). Further research might also include study of the recently identified association between the cyclic alternating pattern (CAP) in patients with fibromyalgia and poorer quality of sleep. CAP is a measure of sleep microstructure, which corresponds to a prolonged oscillation of the arousal level between two reciprocal functional states, phase A (greater arousal) and phase B (lesser arousal) [36]. The pattern represents a condition of instability of the level of vigilance that manifests the brain's fatigue in preserving and regulating the macrostructure of sleep. A higher CAP rate is associated with a greater degree of physical impairment in fibromyalgia [37]. It would be interesting to look beyond the α-EEG anomaly and examine the association between sleep microstructure and attachment style.

## Conclusion

We present the first evidence that a pattern in close interpersonal relationships, particularly anxious insecurity about intimate relationships, is associated with a biological measure of sleep disturbance, the α-EEG anomaly, an anomaly which is associated with significant health problems.

## Abbreviations used

ANOVA analysis of variance, CAP cyclic alternating pattern, df degrees of freedom, ECR-R Experience in Close Relationships – Revised, EEG electroencephalogram, Hz hertz, PSG polysomnographic, REM rapid eye movement, SWS slow wave sleep, SCL Symptom Check List, SD standard deviation

## Competing interests

The author(s) declare that they have no competing interests.

## Authors' contributions

ES was the principal investigator. JH and ES developed the hypothesis and the study method. ES and RM were the primary analysts of the data and the primary authors of the paper. HM developed and validated the method of EEG analysis and trained the sleep technician. All authors discussed the findings, planned and reviewed the paper and approved its final version.
